# Twins' and Singletons' Linguistic Environment: A Systematic Review

**DOI:** 10.3389/fpsyg.2019.02005

**Published:** 2019-09-03

**Authors:** Tommaso Trombetta, Piera Brustia, Lorenzo Curti, Angela M. Caldarera, Eva Gerino, Luca Rollè

**Affiliations:** Department of Psychology, University of Torino, Turin, Italy

**Keywords:** twins, linguistic environment, linguistic input, systematic review, child-directed speech, joint attention, responsiveness, directiveness

## Abstract

**Background:** Among twins, lower linguistic skills emerged when compared with singletons. Considering the association found between parental linguistic input and children's language development, exploring the differences between twins and singletons' linguistic environments could find variables that are potentially associated with the lower linguistic skills of twins.

**Aim:** The current systematic review aims to analyze and systematize the existing literature focused on the comparison of twins' and singletons' linguistic environments within their first 3 years of life. Methodological issues (i.e., the procedure used to assess the linguistic environment, the coding of the linguistic environment's features, the computational method employed to assess the parental linguistic input, and participant characteristics) and differences found among twins and singletons regarding their linguistic environment (i.e., linguistic input quantity, linguistic input complexity, linguistic features of child-directed speech, parental responsiveness, and directiveness, joint attention, and book reading) were highlighted.

**Method:** The Preferred Reporting Items for Systematic Review and Meta-Analysis (PRISMA) statement was followed. Eligible studies were searched through EBSCO, PubMed, and Web of Science. From this search, 1,347 study results emerged, and 8 studies were included.

**Results:** To our knowledge, this is the first systematic review focused on the comparison of twins' and singletons' linguistic environments. Differences between the groups were found in all of the included studies. Data against twins were generally identified regarding all the considered linguistic environment's features. However, conflicting results within and between the included studies emerged, mainly according to the computational method employed (i.e., *twin moms* value, *twin direct dyadic* value, *twin direct dyadic* + *both* value, and input directed toward both children simultaneously).

**Conclusion:** The disadvantaged linguistic environment of twins is likely due to limited parental resources and demands associated with the management of two children of the same age. However, the limited and conflicting data found did not allow for a firm conclusion to be drawn on the differences in the twins' and singletons' linguistic environments. Further studies on the topic are needed.

## Introduction

Several studies found significant differences between twins and singletons regarding their linguistic development (Özçakar et al., 2003; Rutter et al., 2003; Olivennes et al., 2005; Nan et al., 2013; Rice et al., 2014; D'haeseleer et al., 2016). Controlling for potential confounding variables (i.e., age, gender, social background, prematurity), lower language scores among twins were identified. Controlling for social background and excluding children born before 33 weeks of gestation and with neurological or brain damages, a delay of 1.7 months at 20 months of age and a delay of 3.1 months at 36 months of age emerged among twins (Rutter et al., 2003). When comparing twins and singletons in groups matched for age, gender, and parental education, Olivennes et al. (2005) found differences against twins on several dimensions of communication. Similarly, Nan et al. (2013) identified lower scores on communication among twins at ages 3, 6, and 9 months, controlling for prematurity and gender. A recent study confirmed these findings, identifying lower receptive, and expressive linguistic skills among twins compared with singletons matched for age and gender. The results were replicated while even excluding infants born preterm (D'haeseleer et al., 2016). The prevalence of late language emergence found among twins was 38%, while 19.7% emerged within the general population (Rice et al., 2014). Linguistic impairments persisted at least until school age (Rutter et al., 2003; Gucuyener et al., 2011) and were highlighted as differences against twins at 12 years of age (D'haeseleer et al., 2016). However, controlling for birthweight, Dezoete and MacArthur (1996) did not find differences among twins and singletons regarding quality of language and intelligibility of speech. From their perspective, the lower scores that emerged in other studies could be influenced by the overrepresentation within twins' groups of children of low birthweight, a condition which represents about 60% of twin births (Martin et al., 2015). Furthermore, assessing linguistic differences within a triadic context in the home environment, Tremblay-Leveau et al. (1999) found a greater quantity and quality of communication among twins aged 23 months as compared with singletons. The results showed how a triadic setting could represent a favoring context for twins to express their communicative skills during their early life.

Linguistic impairment during the first 3 years of age was associated with concurrent lower social skills (Longobardi et al., 2016) and subsequent linguistic difficulties at 7 (Rice et al., 2008), 8 (Domsch et al., 2012), 13, and 17 years of age (Rescorla, 2005, 2009). Children with language impairment showed lower scores on measurements of school readiness (Justice et al., 2009) and academic achievement (van Noort-van der Spek et al., 2012), as well as higher rates of learning disabilities (Young et al., 2002). Behavioral and social problems at 12.5 years of age were found as well (Beitchman et al., 1996).

According to the social interactionist perspective (Snow, 1972), which emphasizes the environmental role and the value of daily interactions for language development, the linguistic environment's features were widely explored and were found to be predictors of children's linguistic skills (Mol et al., 2008; Farrant and Zubrick, 2012; Rowe, 2012; Weisleder and Fernald, 2013; Levickis et al., 2014; Tamis-LeMonda et al., 2014; Hudson et al., 2015; Sandbank and Yoder, 2016; Conway et al., 2018; Paavola-Ruotsalainen et al., 2018; Smith et al., 2019). Within the twin population, several characteristics of the linguistic environment were explored and compared with those of singletons to highlight variables potentially associated with the lower linguistic skills found among twins: input quantity, input complexity, child-directed speech (CDS) linguistic features, parental responsiveness and directiveness, joint attention (JA), and book reading. However, limited and conflicting results were found (Lytton et al., 1977; Conway et al., 1980; Bornstein and Ruddy, 1984; Tomasello et al., 1986; Stafford, 1987; Ostfeld et al., 2000; Butler et al., 2003; Thorpe et al., 2003). CDS refers to a specific linguistic pattern directed toward children, which is different in its features from the register used to communicate with adults (Golinkoff et al., 2015). CDS is characterized by the use of an exaggerated intonation, a simple structure, short and repetitive utterances, and a high frequency of questions and other forms of linguistic interaction (e.g., imitations, recasts, and expansions) that are useful to promote the flow of conversation. These features allow adults to attract the child's attention and make the language learning process easier (Ratner, 2013; Gonçalves Barbosa et al., 2016; Suttora et al., 2017).

In the general population, several characteristics of CDS were found to be associated with children's linguistic skills. First, the quantity of the input provided by parents emerged as a relevant factor (Hurtado et al., 2008; Rowe, 2012; Weisleder and Fernald, 2013). A positive association was found between the number of *word tokens* and utterances produced by mothers during the first 19 months and the children's vocabulary and efficiency in spoken language understanding at 24 months (Hurtado et al., 2008; Weisleder and Fernald, 2013) and 30 months (Rowe, 2012)

In addition to input quantity, the complexity of CDS influences language development as well (Hoff and Naigles, 2002; Sandbank and Yoder, 2016). A positive association was found between mean length of utterances (MLU) and the subsequent children's vocabulary production (Hoff and Naigles, 2002). However, a recent meta-analysis found only a weak positive association between length of parental input and language development in children with disabilities (Sandbank and Yoder, 2016). Longer utterances likely provide greater grammatical complexity and richer information regarding new words, which could be useful to children to better understand the input meaning and build a stronger vocabulary (Hoff and Naigles, 2002; Sandbank and Yoder, 2016). Nonetheless, the benefits of greater input complexity could vary on the basis of children's linguistic skills (Sandbank and Yoder, 2016).

In addition, parental responsiveness and directiveness were shown to be related, in opposite directions, with the children's linguistic skills (Murray and Hornbaker, 1997; Paavola et al., 2005; Levickis et al., 2014; Hudson et al., 2015; Conway et al., 2018; Paavola-Ruotsalainen et al., 2018; Smith et al., 2019). Parental responsiveness refers to parenting behaviors and communicative acts that follow linguistic input and actions produced by the child (Paavola et al., 2005; Tamis-LeMonda et al., 2014). By increasing the child's involvement, responsiveness promotes parent-child communication and the availability of resources that are useful to learn new linguistic skills (Hudson et al., 2015). Accordingly, responsiveness was found to be associated with comprehensive skills at 12 months (Paavola et al., 2005; Paavola-Ruotsalainen et al., 2018) and with receptive and expressive skills at 24, 36 (Levickis et al., 2014), and 48 months (Hudson et al., 2015).

On the other hand, directiveness is characterized by the parental inclination to redirect the infant's attention to control the child's behavior (Murray and Hornbaker, 1997; Smith et al., 2019). Several studies identified a negative association between parental directiveness and children's receptive and expressive language skills at 24 (Murray and Hornbaker, 1997), 36, and 48 months (Conway et al., 2018; Smith et al., 2019).

Moreover, joint attention (JA) was identified as an influencing factor for children's linguistic skills. JA refers to interactions where the parent and child share their attentive focus toward the same object (Akhtar and Gernsbacher, 2007; Farrant and Zubrick, 2012). JA interactions allow the child to understand the reference of the parent's communication, increasing his or her opportunities to learn new words and improving their appropriate use (Scofield and Behrend, 2011). In line with these considerations, the quantity of time mother and child spent in JA interaction was found to be positively associated with receptive and expressive language skills during the first 3 years of life (Saxon, 1997; Markus et al., 2000; Farrant and Zubrick, 2012).

Lastly, parent-child book reading also represents a positive learning opportunity by providing occasions for learning new words within a stimulating context (Mol et al., 2008; Farrant and Zubrick, 2013; Salo et al., 2016). A meta-analysis conducted by Mol et al. (2008) identified an association of moderate effect size between dialogic book reading and expressive vocabulary, as well as an association of small effect size with receptive vocabulary.

Socioeconomic status (SES) is an important factor as well. Children at the lower levels of SES experience a lessened quantity and quality of linguistic input (Schwab and Lew-Williams, 2016; Inglebret et al., 2017). Specifically, Hoff (2003) highlighted the mediation role of the linguistic environment on the association between SES and the child's linguistic development. The author showed how SES impacts the quality of the linguistic environment experienced by the child, which in turn influences the child's linguistic development. Despite the limited evidence that twins are born in low SES families or contexts, it is possible that their birth influences the overall income of the family in comparison to a singleton birth. McKay (2010) showed that twins were commonly born in families with a low SES. Thus, it is important to explore further the association between SES and language development in twins, controlling for SES when assessing linguistic differences among twins and singletons.

In sum, CDS quantity and quality, maternal responsiveness and directiveness, JA interactions, and parent-child book reading emerged as relevant factors involved in the language development of children, particularly during the first 3 years of life. Despite these findings, limited studies have explored the association between language development and the linguistic environment's features within the twin population. To our knowledge, only five studies explored the association between parental linguistic input and twins' linguistic skills: a relation between the child's language development and the number of maternal words or utterances (Conway et al., 1980; Tomasello et al., 1986; Stafford, 1987; Ostfeld et al., 2000), CDS features (Tomasello et al., 1986; Stafford, 1987), joint attentional interactions (Tomasello et al., 1986), indicators of responsiveness and directiveness (Stafford, 1987) and the maternal engagement in dialogic book reading with the child (Thorpe et al., 2003) emerged. However, different computational methods were employed by these studies to assess twins' linguistic environment features, and potential confounding variables were not controlled for in most of the studies as well (e.g., SES, gender, birthweight. and prematurity). Considering the small number of data available and the methodological limitations identified, the findings emerged do not allow for a firm conclusion to be drawn and further studies are needed.

Considering these preliminary data, exploring the differences in the linguistic environment of twins and singletons could be particularly relevant in highlighting factors that are potentially associated with the lower linguistic skills emerging among twins.

## Aim

The aim of our paper is to review the existing literature focused on the comparison of the linguistic environment of twins and singletons within the first 3 years of life, when environmental features emerged as critical factors for language development as discussed in the section Introduction. Moreover, we will systematize the methodological features of the studies included and the differences that have emerged between the groups to highlight factors potentially associated with the poorer linguistic skills found among twins.

Specifically, in the current systematic review, we aim to explore the following differences among twins and singletons regarding the linguistic environment's features, which, according to the results from the literature, are relevant for the child's linguistic development: number of words or utterances, linguistic features of CDS, parental responsiveness and directiveness, JA interaction, and parent-child book reading. Furthermore, we identify the differences between the studies included regarding the procedure used to assess the linguistic environment, the coded linguistic environment's features, the computational method employed to assess the parental linguistic input, and the characteristics of the groups included as participants.

## Method

The current systematic review was conducted using the PRISMA guidelines (Table 1; Moher et al., 2009). We referred only to published data; therefore, the study did not require the approval of the Ethical Scientific Committee.

**Table 1:** Studies included in the systematic review.

**References**	**Title**	**Journal**	**Study design**	**Sample or participants**	**Procedure**	**Differences in linguistic environment**
Lytton et al., 1977	The impact of twinship on parent-child interaction	Journal of Personality and Social Psychology	Observational study	46 pairs of same sex male twins, 44 male singletons (with sibiling), and respective parents. Mean age: 32,4 months	Home observation of unstructured interaction (coded with the Parent-Child Interaction Code PACIC). Parental language measures: rate of mother-child speech per minute and rate of father-child speech per minute	Mothers and fathers of singletons speak more to their children than parents of twins
Conway et al., 1980	Twin-singleton language differences	Canadian Journal of Behavioral Science	Observational study	12 set of twins, 24 singletons, and respective mothers. Age: 32–33 months	Home observation. Maternal speech measures: complexity (based on four measures: subject phrase, predicate phrase, verb complexity, and additional points), rate of speech per minute overall, and rate per minute of mother-to-child speech.	Significant differences against twins in Rate Mother-to-Child and in the complexity score.
Bornstein and Ruddy, 1984	Infant attention and maternal stimulation: predictor of cognitive development in singleton and twins	Attention and Performance X: Control of Language Processes. Editet by: Herman Bouma and Don G. Bouwhus	Observational and longitudinal study	20 singleton, 11 twin pairs and respective mothers. Age: 4 months (first assessment) and 12 months (second assessment)	Home observation of two maternal activities: encouraging the babie's attention to stimuli in the environment verbally and physically, and talking to the baby.	At 4 months mothers of twins encourage each baby's attention to the environment less than half as often on average as do mother of singletons, and talk to them less than mothers of singleton talk to their children. Maternal differences are stable. At 12 months twins' mothers encourage baby's attention 60% as often as mothers of singletons and vocalize 50% as often
Tomasello et al., 1986	Linguistic environment of 1- to 2- years old twins	Developmental Psychology	Observational and longitudinal study	6 sets of twins, 12 singletons, and respective mothers. Age: 15 (first assessment) and 21 months (second assessment)	Home observations. Parental language evaluated: (1) joint attention: For each interaction, it was established: the initiator; the following response (no response, a brief response, or a relatively extended period of joint attentional focus on some object or event); and who primarily maintained the state of joint attentional—the mother (mother lead), the child (child lead), or equally by both (equal lead). Joint attentional interaction with both twins and the mother was coded as a three-way joint interaction. (2) Child-directed speech: number and mean length (MLU) of child-directed utterances; their proportional distribution into comments, directives, and questions; proportion of utterances characterized by “motherese” intonation. For the twins, mother utterance was coded for its address, with utterances directed to both children simultaneously coded as both. Maternal use of an object word in an utterance directed to the child (or both twins) coded for whether it occurred in a directive or a non-directive form and if the mother used gestures to refers to the object. (3) Conversational responses (only at 21 months): conversation maintaining: imitation, recast, acknowledgment, and topic continuation. Conversational flow disruption: ignore, request for clarification and topic change.	Three computational method employed: (a) “twin moms” value; (b) “twin direct dyadic” value; (c) “twin direct dyadic + both” value. (1) Joint attention: employing the “twin moms” twins and their mothers initiated more social interactions than singletons; with the “twin direct dyadic” value initiated fewer interactions. Regardless the computational method used, twins spent less time in joint attention interactions, and twins and their mothers engaged in a much higher proportion of mother lead joint interactions, in a lower proportion of equal lead joint interactions and in no child lead joint interactions. There were no child age effects or Child Age X Birth Status interactions. (2) Child directed speech: with the individually based twin values, twins had fewer utterances directed to them, and these utterances were of shorter average length (MLU). Regardless the computational method used, twins received a higher proportion of directive utterances and a lower of comments and questions. The proportion of child-directed utterances referring to objects was higher than that of the singletons using the “twin direct dyadic” value. Regardless the computational method used twin mothers referred to objects almost exclusively in directive utterances and almost never in non-directive utterances. From T1 to T2 the proportion of utterances with a motherese intonation declined for all children (using all three values); The MLU of utterances stayed roughly the same for the twin children whereas it rose for the singleton children(using both individually based values); singletons showed a rise in the proportion of directives and a decline in the proportion of comments, while the proportion of questions rose over time for all children (using all three values). (3) Conversational responses: Twin mothers used imitation more often and topic continuation less often.
Stafford, 1987	Maternal input to twins and singleton children: implication for language acquisition	Human Communication Research	Observational study	22 mothers of twins and 22 mothers of singletons (with older sibling). Mean age of twins: 28 months and 16 days; mean age of singletons: 28 months and 15 days	Laboratory observation. The transcripts were divided into utterances, then coded for: (1) Discourse feature: imitations; expansions; extensions; items related to action; topic continuations; semantically unrelated utterances; yes-no answers; synergistic sequences; maternal self-answers; unintelligible remarks; fragments; unclassified utterances; each discourse feature was coded for its direction. Total frequency of utterances in each category regardless of direction was computed. (2) Illocutionary force (exploring two areas: responsiveness/eliciting and controlling/directing): commands (direct and indirect), repairs, questions, positive and negative acknowledgments, prompts, attention devices, spontaneous declaratives, and unclassified utterances. Each illocutionary force feature was coded for its direction. Total frequency of utterances in each category regardless of direction was computed. (3) Conversational style: number of utterances produced by the mother and children; total number of maternal utterances; number of utterances directed toward the target child individually and toward both children simultaneously; maternal self-utterances; number of utterances produced by the target child; number of utterances produced by both children; ratio of maternal utterances to the number of utterances produced by both children.	(1) Discourse features: (a) target child: more imitations, expansions, extensions, items related to actions, and maternal self- repetitions were produced by singletons' mothers. (b) Both children: twins' mothers used more imitations, extensions, utterances related to actions, topic continuations, semantically unrelated utterances, maternal self-repetitions, yes/no answers, and stock expressions. (c) Total environment: singletons' mothers produced more extensions, utterances related to actions, and stock expressions. (2) Illocutionary features: (a) target child: singletons' mothers produced more questions, positive acknowledgments attention devices, and spontaneous declaratives. (b) Both children: twins' mothers used significantly more commands, questions, positive acknowledgments, attention devices, and spontaneous declaratives. (c) Total environment: more commands, repairs and unclassified remarks were produced by twins' mothers. Singletons' mothers used more questions. (3) Style Parameters: singletons' mothers addressed more utterances toward the target children. Twins' mothers produced more utterances directed toward both children simultaneously and talked more to themselves. The ratio of maternal utterances to target child utterances was ~3 to 1 in the singleton environment and 4.5 to 1 in the twin environment. The ratio of maternal utterances to the number of utterances produced by both children was about 1.5 to 1 in the singleton environment and 2.3 to 1 in the twin environment.
Ostfeld et al., 2000	Maternal behavior toward premature twins: implications for development	Twin Research	Observational and longitudinal study	8 premature twins, 22 premature singletons, and respective parents. Age: 1 (first assessment) and 8 months (second assessment)	Home observation (coded with the Modified Beckwith mother-Infant behavior checklist). Maternal behavior measured: positive verbalization (unprompted or responsive to)	Unprompted by and in response to the child, singletons' mothers more likely talk to their children; both groups maintained its performance (from T1 to T2).
Butler et al., 2003	Maternal speech style with prelinguistic twin infants	Infant and Child Development	Observational study	21 mothers of twins and 21 mothers of singletons. Age: 4 months	Videotaped Still-Face procedure. Maternal speech was coded for: (1) speech focus: Infant focus, Mother-Focus, and Other-Focus; (2) content/complexity subcategory: subcategories of Infant-Focus speech: Description, Responsive, Conversation, Simple Repetition, Semantic Repetition, Agency. Subcategories of Mother-Focus speech: Prompt, Game, Song, Description, Self-Reference; (3) syntax subcategory: Interrogative, Declarative, Imperative, Contentless; (4) Presence/Absence of negativity.	(1) Singletons' mothers produced a higher proportion of Infant-Focus utterances; (2) Sub-categories of Infant-Focus: singletons' mothers used a higher proportion of utterances that ascribed agency to the infant and more responsive utterances. (3) Syntax: singletons' mothers produced more Interrogatives; twins' mother used more declaratives.
Karen Thorpe, Michael Rutter and Rosemary Greenwood	Twins as a natural experiment to study the cause of mild language delay: II: Family interaction risk factors	Journal of Child Psychology and Psychiatry	Observational and longitudinal study	96 twin pairs, 98 pairs of singletons, and respective mothers. Age: 20 (first assessment) and 36 months (second assessment)	Home observations of an unstructured (at 20 months) and two structured (reading of a novel picture book and playing with a toy) interactions (at 20 and 36 months). The set of behaviors coded during the observation is not completely available.	At 20 months twins' mother more likely addressed the two children as a pair rather than individually. Toy observation: twins' mothers less likely provided strong motivation to the child. Book observation: twins' mothers less likely engaged the child in elaborations while looking at pictures, invited the child to say something, appeared familiar reading to the child, and reported regular book sharing (meaning getting the child to look at books and talk about the pictures or point to them). At 36 months, on the book interaction there was no longer a difference on mother motivating children, or in elaborating, or in inviting the child to talk.

## Data Source and Search Strategy

Two independent reviewers searched in titles, abstracts, and full texts through EBSCO (CINAHL Complete, eBook Collection [EBSCOhost], Education Source, ERIC, Family Studies Abstracts, Gender Studies Database, Historical Abstracts with Full Text, Mental Measurements Yearbook, PsycARTICLES, PsycINFO, Race Relations Abstracts, Social Sciences Abstracts [H. W. Wilson], Sociology Source Ultimate, and Violence & Abuse Abstracts), PubMed, and Web of Knowledge to find eligible studies. Considering the limited amount of data available, we did not impose a time limit for papers searching, and we searched for both papers and books published from the beginning to May 2019. The following keywords were used: (“twin^*^” or “multiple birth^*^”) AND (“IDS,” or “infant directed speech,” or “CDS,” or “child-directed speech,” or “child addressed speech,” or “infant addressed speech,” or “motherese,” or “baby talk,” or “linguistic environment,” or “maternal speech”, or “paternal speech,” or “parental speech”, or “speech input,” or “language input,” or “linguistic input,” or “maternal input,” or “paternal input,” or “parental input,” or “JA,” or “joint attention,” or “responsive^*^,” or “directive^*^,” or “book reading”, or “mother child^*^ interact^*^,” or “father child^*^ interact^*^,” or “parent child^*^ interact^*^,” or “mother infant interact^*^,” or “father infant interact^*^,” or “parent infant interact^*^”).

## Inclusion And Exclusion Criteria

The following criteria were used for the inclusion of studies in the systematic review: (a) the comparison of twins' and singletons' linguistic environments, (b) occurring within the children's first 3 years of life, and (c) the use of the English language in the papers. Studies that did not match these inclusion criteria were excluded. Furthermore, papers or book chapters that included triplets, quadruplets, or higher order multiples were excluded on grounds that they considered a different population that was not the focus of the current review. Research studies employing a composite measure of the parental environment that included an assessment of linguistic features but did not allow for the extraction of specific features from the parental linguistic input were excluded because they do not enable a comparison of the provided linguistic input.

## Study Selection And Data Extraction

**Figure 1:**
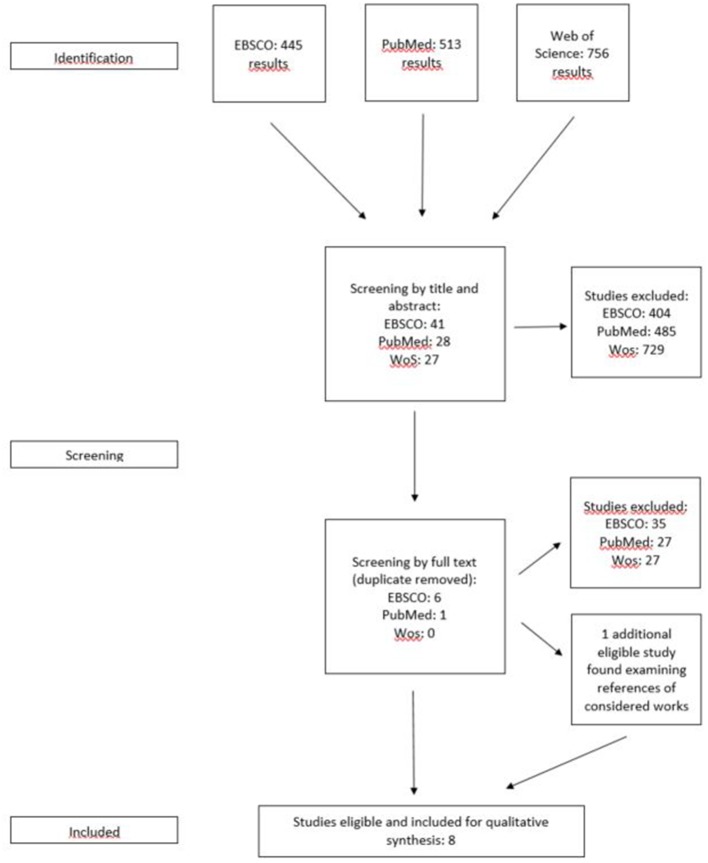
Flow diagram of the selection procedure.

From the 445 papers that resulted from a first search on EBSCO, 41 were selected for the full text review; from PubMed's 513 results, 28 were selected, and from Web of Knowledge's 756 results, 27 papers were selected. It is noteworthy that the majority of the studies found on the three databases focused on the use of twins as a study method rather than as a specific population, were oriented to the study of genetics, and involved several conditions in the shared and non-shared environmental factors. A large number of papers were excluded from the full text review primarily due to this reason. After removing duplicates, the full text review left only seven papers eligible, which were included in our systematic review. From examining references in the selected papers, one more book chapter was identified and added. Overall, seven papers and one book chapter were included in the current paper (see Figure 1). Two independent reviewers conducted data extraction, and discrepancies were discussed to obtain a consensus.

## Results

In the next section, the methodological issues identified within the studies are explored to review the following: the procedure used to assess the linguistic environment, the coded linguistic environment's features, the computational method employed to assess the parental linguistic input, and the characteristics of the groups included as participants. Subsequently, the differences found within and between the reviewed research reviewed will be highlighted regarding the linguistic environment of twins and singletons, specifically focusing on the following: quantity of linguistic input, CDS linguistic features, parental responsiveness and directiveness, JA interaction, and parent-child book reading.

## Methodological Issues

First, although all the papers adopted an observational approach, two papers used a laboratory setting to assess the linguistic environment of the subjects involved (Stafford, 1987; Butler et al., 2003), and six studies employed a naturalistic setting, conducting the observation in family homes (Lytton et al., 1977; Conway et al., 1980; Bornstein and Ruddy, 1984; Tomasello et al., 1986; Ostfeld et al., 2000; Thorpe et al., 2003). Note that the use of a laboratory setting to assess the linguistic features of the family imposes the presence of a camera and does not consider the common demands of the home environment; both of these factors can influence parent-infant interactions (Stafford, 1987; Butler et al., 2003).

Regarding the linguistic environment's coded features, six studies assessed the number of words or utterances directed toward the children (Lytton et al., 1977; Conway et al., 1980; Bornstein and Ruddy, 1984; Tomasello et al., 1986; Stafford, 1987; Ostfeld et al., 2000). Two studies employed an assessment of the parental input complexity (Conway et al., 1980; Tomasello et al., 1986): one study assessed MLU (Tomasello et al., 1986), and one study employed a complexity composite measure based on the score obtained on four measures: subject phrase, predicate phrase, verb phrase complexity, and additional points (Conway et al., 1980). Three studies assessed linguistic features of CDS; however, the studies varied greatly on the variables coded (Tomasello et al., 1986; Stafford, 1987; Butler et al., 2003). Three studies evaluated characteristics of parental responsiveness and directiveness (Tomasello et al., 1986; Stafford, 1987; Butler et al., 2003). Moreover, three research studies assessed respective JA features, that is, the mother's propensity to encourage the infant's attention to the environment (Bornstein and Ruddy, 1984), JA interactions (Tomasello et al., 1986), and attention devices used (Stafford, 1987). Lastly, one study evaluated maternal input during unstructured activity and two structured activities (playing with toys and book reading) (Thorpe et al., 2003). Although observation of the family interaction was present in all the papers, differences emerged in the computational method used to assess the parental linguistic input. Tomasello et al. (1986) defined three different computational methods: the *twin moms* value, which counts the total communication produced by the mothers regardless of the direction and compares the input provided by twins' and singletons' mothers; the *twin direct dyadic* value, which considers the communication directed only toward the target twin; and the *twin direct dyadic* + *both* value, which instead codes the communication directed toward the twins pair contemporaneously as communication addressed to the twin target of the study; both the twin direct dyadic value and the twin direct dyadic + both value compared linguistic input toward twins with the communication directed toward the singleton individually. These values highlight different considerations about the input assumed as relevant for child development. The use of a twin direct dyadic value emphasizes the major role of the linguistic input directed exclusively to the child, whereas the adoption of the twin direct dyadic + both value implies the consideration of the communication directed toward both children as relevant for the infant's language development. With this classification as reference, we can affirm that one study in the current review employed the twin direct dyadic + both value (Lytton et al., 1977), while three papers adopted a mixed method (Conway et al., 1980; Tomasello et al., 1986; Stafford, 1987). Conway et al. (1980) used the twin direct dyadic value and the twin direct dyadic + both value; Stafford (1987) employed the twin moms value and the twin direct dyadic value, adding furthermore an assessment of the communication exclusively directed toward both children simultaneously (both for twins and singletons, including a singleton group with siblings). Only the study by Tomasello et al. (1986) used all three values mentioned above. Butler et al. (2003) were the only ones who adopted a process of observation that did not include both the twins in the interaction and coded only the communication directed toward the target child involved. Bornstein and Ruddy (1984), Ostfeld et al. (2000), and Thorpe et al. (2003) did not provide clear information; for this reason, we were not able to classify the computational method used.

Some differences can be identified regarding the groups included as participants: five papers used sets of twins that were compared with singletons with no siblings, that is, comparing a triadic situation with a dyadic situation (Conway et al., 1980; Bornstein and Ruddy, 1984; Tomasello et al., 1986; Ostfeld et al., 2000; Butler et al., 2003). Three studies compared twins and singletons with siblings in order to replicate the same family structure of twins' families (Lytton et al., 1977; Stafford, 1987; Thorpe et al., 2003). This methodological solution sought to understand if the differences found between the groups were actually due to factors exclusively related to the twin situation and not only to the demands associated with the presence of two children simultaneously. Three studies assessed children at the prelinguistic age of 4–8 months (Bornstein and Ruddy, 1984; Ostfeld et al., 2000; Butler et al., 2003), while five research studies considered children between 15 and 36 months of age (Lytton et al., 1977; Conway et al., 1980; Tomasello et al., 1986; Stafford, 1987; Thorpe et al., 2003), when infants are already starting to produce words (Taylor et al., 2018).

Furthermore, while most of the studies included controlled for age (Lytton et al., 1977; Bornstein and Ruddy, 1984; Tomasello et al., 1986; Stafford, 1987; Ostfeld et al., 2000; Butler et al., 2003; Thorpe et al., 2003) and gender (Conway et al., 1980; Tomasello et al., 1986; Stafford, 1987; Ostfeld et al., 2000; Butler et al., 2003) when assessing differences between the twins' and singletons' linguistic environments, only four studies controlled for prematurity (Bornstein and Ruddy, 1984; Stafford, 1987; Ostfeld et al., 2000; Butler et al., 2003) and three for birthweight (Tomasello et al., 1986; Stafford, 1987; Ostfeld et al., 2000), variables that emerged as potential confounding variables (Dezoete and MacArthur, 1996; Rutter et al., 2003; Olivennes et al., 2005; Nan et al., 2013; D'haeseleer et al., 2016). Moreover, it is noteworthy that only three studies controlled for SES (Conway et al., 1980; Tomasello et al., 1986; Butler et al., 2003). Considering the influence of SES on both the parental linguistic input and the children's linguistic skills found within the general population (Hoff, 2003; Schwab and Lew-Williams, 2016; Inglebret et al., 2017), as well as the preliminary data regarding the negative association between twin births and SES (McKay, 2010), further studies would need to control for this variable.

Lastly, four studies observed the characteristics of the linguistic environment at two time points (Bornstein and Ruddy, 1984; Tomasello et al., 1986; Ostfeld et al., 2000; Thorpe et al., 2003), while four research studies assessed parental input at only one time point (Lytton et al., 1977; Conway et al., 1980; Stafford, 1987; Butler et al., 2003).

## Linguistic Environment Differences Between Twins And Singletons

All the studies eligible for the current systematic review showed significant differences between twins' and singletons' linguistic environments (Lytton et al., 1977; Conway et al., 1980; Bornstein and Ruddy, 1984; Tomasello et al., 1986; Stafford, 1987; Ostfeld et al., 2000; Butler et al., 2003; Thorpe et al., 2003) and data against twins generally emerged. However, conflicting results within and between the studies mainly based on the computational method employed. For this reason, the results obtained do not allow for firm conclusions about the differences in the linguistic environments of twins and singletons.

## Linguistic Input

### Linguistic Input Quantity

The six studies interested in the twins' and singletons' differences in the number of words or utterances expressed by parents showed results in favor of the singletons group (Lytton et al., 1977; Conway et al., 1980; Bornstein and Ruddy, 1984; Tomasello et al., 1986; Stafford, 1987; Ostfeld et al., 2000). However, differences emerged according to the computational method used. Employing the twin moms value, Tomasello et al. (1986) and Stafford (1987) did not find significant differences between groups. On the other hand, employing the twin direct dyadic value (Conway et al., 1980; Tomasello et al., 1986; Stafford, 1987) and the twin direct dyadic + both value (Lytton et al., 1977; Conway et al., 1980), significant results against twins emerged. The only study that computed the utterances directed toward both children simultaneously highlighted instead a larger number of words within the group of twins (Stafford, 1987). The results described showed no differences regarding the number of words or utterances computed with the twin moms value (Tomasello et al., 1986; Stafford, 1987). Otherwise, considering the number of words/utterances addressed to the child target of the study, computed both by considering exclusive input toward the target child or adding input directed to the pair simultaneously, significant differences against twins emerged in all the studies (Conway et al., 1980; Tomasello et al., 1986; Stafford, 1987). Twins' mothers do not speak less compared with singletons' mothers, although they talk less to the target child (also adding input addressed to the pair) in comparison with singletons' mothers (Conway et al., 1980; Tomasello et al., 1986; Stafford, 1987). The origin could be due to the nature of the twin situation and the limited attentive resources that they can direct toward two children of the same age (Conway et al., 1980; Tomasello et al., 1986).

### Linguistic Input Complexity

Regarding the complexity of the linguistic environment provided by parents, results against twins generally emerged. Lower MLU among twins' mothers was shown by Tomasello et al. (1986). Significant differences were highlighted by exclusively employing the twin direct dyadic (results were not replicated controlling for birthweight) and the twin direct dyadic + both values, whereas no differences emerged using the twin moms value. In addition, Conway et al. (1980) found a reduced language complexity in the twins' linguistic environment, assessing a complexity composite measure based on the score obtained on four measures: subject phrase, predicate phrase, verb phrase complexity, and additional points (i.e., negative expressions, conjunctions, and questions).

### Linguistic Features of Child-Directed Speech

Considering the results found in the studies that assessed the linguistic features of CDS, generally the disadvantaged condition of twins emerged (Tomasello et al., 1986; Stafford, 1987; Butler et al., 2003). However, conflicting findings surfaced according to the computational method used. All three studies showed a reduced number or proportion of questions among the twins' mothers, regardless of the computational method used [note that in the Tomasello et al. (1986) study, controlling for birthweight and child's language skills, the results were not replicated using the twin direct dyadic value] (Tomasello et al., 1986; Stafford, 1987; Butler et al., 2003). Only the Stafford (1987) study, computing the utterances toward both children, highlighted a higher number of questions among this group. The assessment of the proportion or number of utterances aimed at the topic continuation—parental linguistic features that ensure the flow of the conversation as questions (Tomasello et al., 1986)—showed diverging results. Tomasello et al. (1986) highlighted a reduced proportion among twins, whereas Stafford (1987) found differences only when considering input directed toward both children simultaneously, showing a higher number of topic continuation utterances among twins. Moreover, the author highlighted the lower participation of twins in the conversation compared with singletons, which is a condition that represents the mother's attempt to control and limit the conversation (Stafford, 1987). A higher number of declaratives—utterances with the function to assert or describe and which characterize the adult-directed speech (Butler et al., 2003)—were found among twins by Butler et al. (2003). On the other hand, conflicting results were found in the Stafford (1987) study according to the computational method used. Employing the twin direct dyadic value, the author found a lower number of spontaneous declaratives among twins, while a higher number was found considering the utterances directed toward both children simultaneously. Considering the remaining differences in kinds of utterances, which represent a facilitative/non-facilitative linguistic environment, the results highlighted the unfavorable condition of twins. Coding the linguistic input addressed toward both children simultaneously, Stafford (1987) found more semantically unrelated utterances and yes/no answers among twins, as well as more repairs and unclassified remarks adopting the twin moms value—all utterances representative of a non-facilitative linguistic environment. Computing input with the twin direct dyadic value, Stafford (1987) found a lower number of positive acknowledgments, while Tomasello et al. (1986) identified no differences. Positive acknowledgments are representative of an adaptive linguistic environment; expressing approval for what the child says then increases the child's linguistic confidence (Stafford, 1987). Computing the utterances directed toward both children simultaneously, Stafford (1987) also identified a larger number of maternal self-utterances among twins' mothers. Lastly, Tomasello et al. (1986) found a larger use of object words among twins' mothers using the twin direct dyadic value, which provides a source of word learning during early development. No differences were found employing the twin direct dyadic + both and the twin moms values.

### Parental Responsiveness and Directiveness

Considering the studies that assessed linguistic input by characterizing responsive and directive interactions, the results highlighted the disadvantaged linguistic environment of twins for several variables (Tomasello et al., 1986; Stafford, 1987; Butler et al., 2003). However, conflicting results emerged on the basis of the computational method used. Butler et al. (2003) identified a lower proportion of infant-focused utterances among the twins' mothers. Regarding the subcategory of infant-focused speech content, mothers of twins showed a lower proportion of utterances conveying agency and responsiveness to the child, which underlines the difficulty in understanding the meaning of the child's cues. This condition entails a non-optimal linguistic environment (Butler et al., 2003). Moreover, using the twin moms and the twin direct dyadic values, fewer extensions and items related to action (both considered responsive speech features) were found among twins' mothers in Stafford (1987). On the other hand, when coding input directed toward both children simultaneously, the opposite result emerged (Stafford, 1987). Tomasello et al. (1986) found a greater proportion of imitations among twins, while Stafford (1987) found the same results (considering the number and not the proportion) by only computing the verbal stimulations directed toward both children simultaneously. On the other hand, using the twin direct dyadic value, Stafford (1987) found a greater number of imitations among singletons and no differences employing the twin moms value. However, the role of imitation is controversial; from Stafford's (1987) perspective, imitations represented the mothers' responsiveness and ability to improve the language learning occasions. In contrast, Tomasello et al. (1986) categorized the imitations as utterances aimed at maintaining the conversation and stated that this linguistic form minimizes the stimulation and limits the speech escalation, highlighting its maladaptive role. It is noteworthy that the statistical analysis employed in these studies to evaluate the correlation between the linguistic environment's features and the children's linguistic development highlighted opposite results, supporting the conflicting theoretical perspective mentioned above (Tomasello et al., 1986; Stafford, 1987). Regarding the utterances that represent a lack of responsiveness, Stafford (1987) highlighted a reduced number of maternal self-answers among twins adopting the twin direct dyadic value, whereas a greater number was found when coding input directed toward both children simultaneously. Using this latter computational method, the results showed a higher number of stock expressions among twins, whereas when employing the twin moms value, the number of stock expressions was higher among singletons (Stafford, 1987).

On the other hand, regardless of the computational method used, Tomasello et al. (1986) highlighted a larger proportion of directive utterances among the twins' mothers, while Stafford (1987) found the same results (in terms of number of commands, not of proportion) only using the twin moms value and computing the input directed toward both children simultaneously. Butler et al. (2003) found no differences between groups (twin direct dyadic value). In the Tomasello et al. (1986) study, regardless of the computational solution adopted, mothers of twins referred to objects mainly with a directive form and almost never with non-directive utterances.

### Joint Attention and Book Reading

Regarding JA interaction, although the results identified the disadvantaged condition of twins for most of the dimensions, the studies showed different results according to the computational method used (Bornstein and Ruddy, 1984; Tomasello et al., 1986; Stafford, 1987). The physical and verbal encouragement of the child's attention toward the environment was higher among singletons' mothers (Bornstein and Ruddy, 1984). In the Tomasello et al. (1986) study, employing the twin direct dyadic value, twins' mothers began fewer JA interactions (results were not replicated controlling for birthweight), whereas using the twin moms value showed opposite results; no differences were found with the dyadic + both value. Moreover, regardless of the computational method used, the authors highlighted a lower number and length of JA interactions, a reduced proportion of equal-led JA interactions, and a higher proportion of JA interactions maintained by the effort of the mother (results were not replicated controlling for birthweight, using the twin direct dyadic value) among twins. Within this group, no JA interaction maintained by the effort of the child (child-led joint interaction) were identified. The use of attention devices to attract the child's attention was higher among twins computing input toward both children simultaneously; the opposite results were found when employing the twin direct dyadic value (Stafford, 1987).

The Thorpe et al. (2003) study, which observed mothers and children involved in an unstructured interaction and two structured interactions (playing with toys and book reading), found that twins' mothers tended to approach the children simultaneously rather than individually (unstructured interaction), showing a reduced probability to motivate the children to action (toy observation) and to involve him or her in the elaboration of pictures and in linguistic production while reading a book. During this latter activity, twins' mothers also appeared less likely to be familiar with reading to the child.

## Longitudinal Studies

Ostfeld et al. (2000) highlighted the same performance and the same differences between groups in the number of verbalization both at T1 (1 month) and T2 (8 months); similarly, Bornstein and Ruddy (1984) found stable differences between T1 (4 months) and T2 (12 months) regarding the encouragement of the child's attention. Tomasello et al. (1986) found no differences between T1 (15 months) and T2 (21 months) in JA interactions but identified a reduction in the proportion of utterances characterized by motherese intonation and an increase in the proportion of questions both in twins and singletons. Only singletons showed a decline in the proportion of comments and a rise in directives (using all three values employed by the respective authors). Furthermore, data identified an increase among singletons on the MLU, while twins showed stable results between T1 and T2: results were obtained, however, using the twin direct dyadic and dyadic + both values and not the twin moms value (Tomasello et al., 1986). Regarding the remaining CDS linguistic features coded, Tomasello et al. (1986) did not find differences between T1 and T2. Thorpe et al. (2003) did not provide clear information on the difference between the first and the second assessment. However, regarding maternal input during the book interaction, the authors did not find the differences between groups at the second assessment (36 months) that they found at the first time point (20 months). According to Thorpe et al. (2003), these data demonstrated how the results obtained at T1 (20 months) are not due to the lack of skills of twins' mothers but are likely related to the demands associated with the presence of twins during the first years postpartum, which affect the relationship and the linguistic environment qualities. From our perspective, these considerations are sustained by the absence of differences between T1 and T2 among twins on the majority of the measures assessed by the studies that considered the first 21 months of life (Tomasello et al., 1986; Ostfeld et al., 2000).

## Discussion

To our knowledge, this is the first systematic review focused on the comparison of twins' and singletons' linguistic environments. Limited data emerged from the literature, only seven papers and one book chapter matched the inclusion and exclusion criteria and were included (Lytton et al., 1977; Conway et al., 1980; Bornstein and Ruddy, 1984; Tomasello et al., 1986; Stafford, 1987; Ostfeld et al., 2000; Butler et al., 2003; Thorpe et al., 2003). Within all the studies included, differences were found between the groups. The results generally showed the disadvantaged condition of twins. Twins' mothers talked less to the target child and provided more non-facilitative input of lower complexity. Furthermore, twins' mothers were less responsive and more directive when interacting with their children, involved their children in fewer and shorter JA interactions, and stimulated their children less during book reading. As stated by several authors, the results against twins are likely due to the demands related to the twin situation, the limited attentive resources available, and the mothers' attempt to control the situation to manage two children of the same age (Tomasello et al., 1986; Stafford, 1987; Butler et al., 2003; Thorpe et al., 2003). The demands that entail the management of two children of the same age emerged from interviews conducted by Holditch-Davis et al. (1999). These findings are in line with the parental difficulties identified in families of twins in the first years of the toddlers' lives (Glazebrook et al., 2004; Olivennes et al., 2005; Sutcliffe and Derom, 2006; Lutz et al., 2012; Beer et al., 2013; Wenze et al., 2015; Anderson et al., 2017). Compared with singletons' parents, twins' parents experienced higher psychological symptoms and parenting stress (Glazebrook et al., 2004; Olivennes et al., 2005; Lutz et al., 2012; Beer et al., 2013; Wenze et al., 2015; Prino et al., 2016). Moreover, they needed greater resources and more involvement to rear twins (Prino et al., 2016). Less optimal interactions among twins and their parents were found in comparison with singletons' families (Glazebrook et al., 2004; Sutcliffe and Derom, 2006; Anderson et al., 2017). However, there were some conflicting results within and between the studies, and the results against twins were not replicated when employing different computational methods (i.e., twin moms value, twin direct dyadic value, twin direct dyadic + both value, and input directed toward both children simultaneously). A need remains to further confirm the results identified and understand the role of the differences found on the child's linguistic skills with specific computational methods to better understand the relevance of the findings against twins for language development. These findings could draw important theoretical and research conclusions about the linguistic environment's features and the input direction that impact twins' linguistic development (i.e., input addressed to the child individually, to the pair concurrently, or expressed by the mother regardless of direction).

On the other hand, it is noteworthy that the results obtained identified data favoring twins for some variables (i.e., use of extensions, items related to action, self-answers, stock expressions, spontaneous declaratives, questions, topic continuation, attention devices, object references, and number of JA interactions initiated by the mother). However, regarding the use of questions, topic continuation, extensions, items related to action, and attention devices, these results were obtained by comparing two triadic situations and coding input directed toward both children simultaneously (Stafford, 1987). These data showed a non-significant negative correlation with the twins' and singletons' linguistic skills (Stafford, 1987). Although the results showed the favoring condition of twins for these dimensions, input directed toward both children simultaneously did not contribute to the target child's language development. These preliminary findings assume theoretical and research relevance, which needs to be further confirmed.

It is also important to note that seven features of the linguistic environment (number of words/utterances, questions, declaratives, directives, topic continuation utterances, imitation, and acknowledgment) were assessed by more than one study. Of these, the results regarding quantity of words/utterances (with the twin direct dyadic value), questions (with the twin direct dyadic and the twin moms values), declaratives (with the twin direct dyadic value), and directives (with the twin moms value) were uniquely replicated by adopting the same computational method, confirming the disadvantaged condition of twins (Conway et al., 1980; Tomasello et al., 1986; Stafford, 1987; Butler et al., 2003).

It is noteworthy that the differences found among the twins' and singletons' linguistic environment are not exclusively due to the comparison of a triadic and a dyadic interaction. Compared with singletons with siblings, twins showed a disadvantaged linguistic environment (Lytton et al., 1977; Stafford, 1987; Thorpe et al., 2003), which could be due to the fact that two children of the same age have the same dependence degree and similar evolutionary needs that can emerge at different times. This entails great demands on the mothers, who cannot rely on the higher independence of one of the children, which would help to limit the double maternal commitment (Tomasello et al., 1986; Thorpe et al., 2003; Wenze et al., 2015). Moreover, twins' mothers were more likely to address the children as a pair, which is a condition that could limit individual stimulation and impact linguistic development (Thorpe et al., 2003). Lastly, as stated by Thorpe et al. (2003), the occurrence of an older sibling rather than a twin could guarantee more complex communication among siblings and a motivation for the mother to adopt a more sophisticated linguistic pattern to comply with the communicative competence of the older child.

Although the results obtained generally showed the disadvantaged condition of twins, conflicting results were identified within and between the studies, mainly according to the computational method employed. The limited data available and the conflicting and not replicated results do not allow the results to be confirmed nor clear conclusions to be drawn regarding the differences in the linguistic environment of twins and singletons.

## Limitations

The current review presents several limitations: First, the results are limited by the reduced number of papers included due to the few studies that comply with the established selection criteria. Second, our review is not a meta-analysis, and this study design does not allow statistical conclusions to be drawn about the results found in the included studies. Lastly, limitations are related to the selection and exclusion criteria used: we excluded those studies that employed measures that—despite the evaluation of the linguistic environment's features—do not provide a clear understanding of the differences among twins and singletons, showing instead composite results of the total environment (Beer et al., 2013; Anderson et al., 2017). Moreover, we selected only studies that compared twins and singletons groups, excluding studies that considered only twins and found results that did not identify disadvantaged linguistic environment's features within the twin population (Barton and Strosberg, 1997; Rendle-Short et al., 2015). Finally, we included only studies published in English, not considering papers published in other languages concerned with the issue, which could provide additional information. The adoption of these selection criteria allowed for a clear identification of the studies concerned with the differences in linguistic environments of twins and singletons, but, on the other hand, did not allow the complexity of the issue to be considered. Other studies that draw conclusions about all the environmental variables involved in linguistic learning would be useful.

## Future Directions

First, considering the limited and conflicting data that emerged, other studies with a comparative design would be useful to further explore the linguistic environment's features (i.e., quantity and complexity of linguistic input provided, linguistic features of CDS, maternal responsiveness and directiveness, JA interaction, and book reading) for which were found differences among twins and singletons in the studies included (Lytton et al., 1977; Conway et al., 1980; Bornstein and Ruddy, 1984; Tomasello et al., 1986; Stafford, 1987; Ostfeld et al., 2000; Butler et al., 2003; Thorpe et al., 2003) and that emerged as influencing factors for language development (Conway et al., 1980; Tomasello et al., 1986; Stafford, 1987; Ostfeld et al., 2000; Thorpe et al., 2003). This would allow for clearer conclusions about the preliminary differences within the studies included in the current systematic review and for further highlighting of factors potentially associated with the lower linguistic skills found among twins.

Furthermore, other studies are necessary to better understand the twins' and singletons' differences according to the computational method used, as well as the relation of these differences with the lower linguistic skills found among twins. Specifically, future studies focused on the comparison of the twins' and singletons' linguistic environments should employ the different computational methods highlighted by Tomasello et al. (1986) (i.e., input addressed to the child individually, to the pair concurrently, or expressed by the mother regardless of direction) to clarify the direction of parental input that entails differences in the twins' and singletons' linguistic environments. Moreover, further studies are necessary to explore the association between the linguistic environment's features against twins (emerged with the specific computational method) and the twins' language development, controlling for variables that could influence the results found (e.g., age, gender, birthweight, prematurity, and SES). These findings could assume a theoretical and research relevance to further confirm the results found in the studies included in the current review and to clarify the input and the input directions that influence twins' linguistic skills. It is noteworthy that only five studies included in the present review—which, to our knowledge, are the only ones in the literature—performed statistical analysis between the twins' linguistic environment and the twins' linguistic skills, finding results that confirm the influence of the linguistic environment's features (Conway et al., 1980; Tomasello et al., 1986; Stafford, 1987; Ostfeld et al., 2000; Thorpe et al., 2003).

Moreover, most of the studies compared a triadic and a dyadic situation (Conway et al., 1980; Bornstein and Ruddy, 1984; Tomasello et al., 1986; Ostfeld et al., 2000; Butler et al., 2003), while only three research studies compared twins and singletons with siblings (Lytton et al., 1977; Stafford, 1987; Thorpe et al., 2003). Further studies that adopt this latter methodological solution are necessary to better understand whether the differences found are actually due to the demands associated with the twin situation—as emerged in the studies included in the current review that compared two triadic contexts (Lytton et al., 1977; Stafford, 1987; Thorpe et al., 2003)—and not only to the comparison of a triadic and a dyadic context and thus to the complexities related with triadic interactions.

Considering that the differences among twins and singletons emerged both at the prelinguistic age and until 21 months of age (Bornstein and Ruddy, 1984; Ostfeld et al., 2000; Butler et al., 2003), with no differences found at 36 months (Thorpe et al., 2003), other studies with a longitudinal design that assess the linguistic environment of twins and singletons until at least age 3 could be useful to understand whether the disadvantaged condition of twins are sustained or resolved as stated by Thorpe et al. (2003).

Lastly, future studies with a cross-cultural design that explore the association between SES and the linguistic environment of twins would improve the level of knowledge of the phenomenon.

## Author Contributions

TT and LR took overall responsibility for the creation of the framework used in this review and the selection of the papers. TT, LC, AC, and EG searched for the articles discussed in the review. LR and PB supervised the entire work. All authors were involved in the discussion, the writing, and the revision of the manuscript, and they gave the final approval of the version to be published.

### Conflict of Interest Statement

The authors declare that the research was conducted in the absence of any commercial or financial relationships that could be construed as a potential conflict of interest.
